# The clinical frailty scale predicts functional decline and mortality when used by junior medical staff: a prospective cohort study

**DOI:** 10.1186/s12877-016-0292-4

**Published:** 2016-06-02

**Authors:** Kate J. Gregorevic, Ruth E. Hubbard, Wen K. Lim, Benny Katz

**Affiliations:** Department of Aged Care, Northern Hospital, 185 Cooper St, Epping, Vic 3076 Australia; North West Academic Centre, University of Melbourne, Melbourne, Australia; Geriatric Medicine Deputy Director, Centre for Research in Geriatric Medicine, The University of Queensland, Brisbane, Australia; Geriatric Medicine, St Vincent’s Hospital, Melbourne, Australia; University of Melbourne, Melbourne, Australia; La Trobe University, Melbourne, Australia; Northern Hospital, Epping, Australia

**Keywords:** Aged, Frailty, Hospitalization, Survival, Frail elderly, Activities of daily living

## Abstract

**Background:**

Increasing frailty is associated with risk of mortality and functional decline in hospitalized older adults, but there is no consensus on the best screening method for use by non-geriatricians. The objective of this study is to determine whether the clinical frailty scale (CFS) can be used to identify patient baseline frailty status in the acute general medical setting when used by junior medical staff using information obtained on routine clinical assessment.

**Methods:**

This was a prospective cohort study in an acute general medical unit. All patients aged 65 and over admitted to a general medical unit during August and September 2013 were eligible for the study. CFS score at baseline was documented by a member of the treating medical team. Demographic information and outcomes were obtained from medical records. The primary outcomes were functional decline and death within three months.

**Results:**

Frailty was assessed in 95 % of 179 eligible patients. 45 % of patients experienced functional decline and 11 % died within three months. 40 % of patients were classified as vulnerable/mildly frail, and 41 % were moderately to severely frail. When patients in residential care were excluded, increasing frailty was associated with functional decline (*p* = 0.011). Increasing frailty was associated with increasing mortality within three months (*p* = 0.012).

**Conclusions:**

A high proportion of eligible patients had the frailty measure completed, demonstrating the acceptability of the CFS to clinicians. Despite lack of training for medical staff, increasing frailty was correlated with functional decline and mortality supporting the validity of the CFS as a frailty screening tool for clinicians.

## Background

Frailty is a state of vulnerability to poor resolution of homoeostasis after a stressor event and is a consequence of loss of reserve across multiple physiological systems which occurs across a lifetime [1]. Frailty can be used to identify older adults who are at increased risk of mortality and functional decline when they are hospitalized [2], but there is no consensus on the most appropriate way for non-geriatricians to identify frailty at the time of hospital admission [3]. Traditionally subjective opinion has been used by non-geriatricians to identify frailty, but this correlates poorly with objective measures of frailty [4].

The frailty phenotype [5] and the frailty index [6] have both been validated against adverse outcomes in large community cohorts. Fried’s frailty phenotype requires measurement of gait speed [5], which is likely to be affected by an acute illness. Comprehensive geriatric assessment (CGA) is a multidimensional patient assessment examining medical, psychological, nutritional, cognitive and functional domains [7]. CGA can decrease mortality and length of stay for hospitalized older adults [7]. The frailty index based on a comprehensive geriatric assessment (FI-CGA) at the time of hospital admission predicts increased risk of mortality and need for residential care [3, 8], but requires geriatrician input. CGA is time and resource intensive and it is not feasible to provide this for all patients who present to hospital at this time. Screening for frailty by non-geriatricians may identify patients most likely to benefit from a CGA [3]. It may also help non-geriatricians with prognostication.

The Clinical Frailty Scale (CFS) [9] was developed to enable frailty to be measured in the outpatient clinical setting [10]. It has demonstrated very good inter-rater reliability [10, 11]. When used by trained assessors it predicts short-term and long-term mortality in acutely hospitalized older adults [11–16] grouped as frail or not frail. A large retrospective cohort study demonstrated that increasing frailty on the CFS has a linear relationship with inpatient mortality and increased length of stay [17]. The CFS is an attractive tool as it can be completed based on routine clinical admission and there is no need for extra equipment, so there are minimal barriers to its’ implementation. Although other frailty measurement tools, such as the Reported Edmonton Frailty Scale (REFS) have been validated against the Geriatrician’s Clinical Impression of Frailty in the inpatient setting, the REFS has features which limit its’ use in patients who do not speak English, or who are hearing or vision impaired [18].

The objective of this study is to determine the predictive validity of the CFS when used by untrained junior medical staff in the acute general medical setting using only routine clinical information.

## Methods

### Subjects and setting

The study took place at St Vincent’s Hospital, a university associated tertiary hospital in inner-Melbourne, Australia. All patients admitted under a general medical unit during August and September 2013, who were aged 65 years or older were included. There were four general medical units consisting of one registrar and two interns, as well as admitting night and day registrars, meaning there were 14 possible candidates to complete the CFS. As the primary focus of the study was to assess utility of the clinical frailty scale, not the doctors, data collection was anonymous to protect staff privacy. Patients were excluded if they were transferred to a different specialty unit. Ethics approval was obtained from St Vincent’s hospital, Melbourne. As the project used data that was collected as part of routine medical care, the ethics committee determined that individual consent was not required.

### Measures and data collection

Copies of the Clinical Frailty Scale (CFS) were placed in the work-rooms of the junior medical staff. These doctors were asked to record a CFS score based on the patient’s functional status prior to admission using only routine clinical assessment. The CFS could be completed at any time during the admission, so patients admitted after hours and on weekends were included. No specific training or incentives were provided. Inter-rater reliability was not measured as this has been demonstrated in prior studies [10, 11] and it was felt that it would apply additional stress to the junior doctors completing the CFS. Demographic data and baseline characteristics were obtained via electronic medical records and chart review (Table 1). Co-morbidities were measured using the Charlson comorbidity index with information from medical records [19, 20].

**Table 1:** Baseline characteristics of study participants

Frailty Score	Number in group	Age (median and range)	Male (%)	Preferred language not English (%)	Home alone/home accompanied/residential care (%)			Charlson Score (mean)
		(*p* = .436)	(*p* = 0.816)	(*p* = 0.184)	(*P* < 0.01)			(*p* = .377)
1-3	29	80 (66-96)	52	48	12 (37)	17 (58)	0	6.4
4-5	68	82 (66-97)	43	56	32 (47)	33 (48)	2 (3)	6.4
6-8	70	83 (66-97)	46	65	15 (21)	31 (44)	24 (34)	6.8
9	3	77 (65-80)	100	100	1 (33)	1 (33)	1 (33)	6.3

The two outcomes investigated were mortality and functional decline. Mortality was measured at three months. This was obtained from hospital records. Functional decline was measured at the time of discharge from the acute hospital. Functional decline was defined as the need for subacute care, determined by a trained nurse assessor and geriatrician, patient being assessed as below pre-morbid function for their activities of daily living or instrumental activities of daily living, or the need for increased services on discharge both of which were determined by allied health practitioners. Patients who died during the admission were excluded from the analysis of functional decline. The information for mortality and functional decline was obtained from hospital records and chart review.

### Analysis

Patients were divided into four groups based on their frailty scores. Patients who were scored at 1-3 on the CFS were defined as not frail (group 1), patients who were scored at 4-5 (group 2) as vulnerable-mildly frail, patients who scored 7-8 (group 3) as moderately to severely frail and patients who scored 9 were terminally ill but not otherwise frail (Group 4). All statistical analysis was performed using Stata version 12.1. The level of statistical significance was set at 0.05. Baseline characteristics between the groups were compared using the chi squared statistic, where applicable (see Table 1). Univariate analysis was performed for all variables (Table 2). Multivariate analysis was performed using two models. All variables which had a *P* value of less than 0.1 were included in the multivariate analysis. Usual residence was also included in a model for functional decline due to theoretical concern regarding confounding.

**Table 2:** Results of univariate analysis

Outcome	Variable	Coefficient	95 % CI	*P* value
Functional decline (exclude all patients in residential care)	Age	0.37	-0.013,0.009	0.702
	Gender	0.081	-0.080,0.244	0.321
	Usual residence	-0.06	-0.195,0.070	0.355
	Charlson score	0.015	-0.020,0.0516	0.386
	Preferred language	-0.00	-0.163,0.161	0.988
Three month mortality	Age	0.001	0.005,0.007	0.756
	gender	-0.111	-0.204,-0.018	0.019
	Usual residence	0.038	-.008,0.085	0.106
	Charlson score	0.016	-0.006, 0.038	0.148
	Preferred language	-0.047	-0.141,0.015	0.316

## Results

### Baseline characteristics

179 eligible patients were admitted during the time period of the study. Average age was 82.0. Frailty scores were obtained for 95 % of patients. 40 % of patients were vulnerable/mildly frail, and 41 % were moderately to severely frail. Only 17 % were not frail (Table 1). There were no significant differences in age, gender or co-morbidity score across the groups. Patients who were more frail were less likely to live home alone (*p* < 0.01). Patients who were moderately to severely frail were most likely to live in residential care (*P* < 0.01). There were no other significant differences in baseline characteristics when grouped by frailty score (Table 3).

**Table 3:** Results of multivariate analysis

Outcome	Model	Number in model	Coefficient	95 % confidence interval	*P* value	OR	95%CI for OR
Functional decline	Univariate	145	0.142	0.033,0.252	0.011	1.8	1.13,2.87
	Model 1	145	0.144	0.035,0.255	0.010	1.82	1.14, 2.91
Mortality and three months	Univariate	169	0.080	0.018, 0.143	0.012	2.5	1.19,5.3
	Model 2	169	0.079	0.017, 0.141	0.012	2.4	1.15,4.97
	Model 3	169	0.061	-0.003,0.126	0.070	2.2	0.098,4.67
	Model 4	169	0.068	0.003,0.133	0.04	2.3	1.15,5.30

Model 1 variables: usual residence, excludes patients in residential care

Model 2 variables: gender

Model 3 variables: gender, Charlson co-morbidity score, usual residence

Model 4 variables: usual residence

### Outcomes

Overall mortality at three months was 11 %. At the time of discharge from the acute hospital, 45 % of patients experienced functional decline. Mean length of stay was 6.7 days, which was not significantly different across frailty groups.

**Fig. 1:**
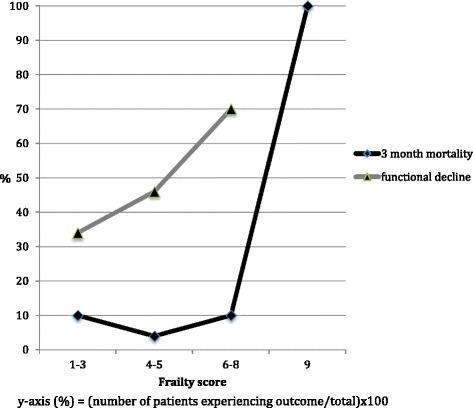
For each increased level of frailty there is a corresponding increase in the percentage of people experiencing functional decline (frailty score 1-3: 34 %, 4-5: 46 % and 6-8: 70 %). There was an overall trend for increasing mortality with increasing frailty (frailty score 1-3: 10 %, 4-5:4 %, 6-8: 10 % and 9: 100 %)

When people who lived in residential care were excluded, in univariate analysis patients who were more frail were also more likely to experience functional decline (OR 1.8, 95%CI 1.13,2.87) (Fig. 1). No other variable was associated with functional decline in univariate analysis. In the multivariate model with usual residence, the association between frailty and functional decline remained significant.

In univariate analysis increasing frailty was associated with increased risk for mortality (OR 2.5, 95 % CI 1.19,5.3) (Table 2). Gender was included in the multivariate model, the OR for this model was 2.4 (95 % CI 1.15,4.97).

## Discussion

This study demonstrates the feasibility of using the Clinical Frailty Scale in the acute general medical setting. The CFS correlates with the important outcomes of death and functional decline. This is the first study the authors are aware of, where no training was provided to junior doctors in order to examine how the CFS functions in a real world setting. This study also shows that this scale is highly acceptable to medical staff as there was a 95 % completion rate. It was completed with information obtained on routine assessment at the time of admission, so the additional workload for junior medical staff was minimal. The combination of acceptability and prognostic guidance supports the role of the CFS as a tool to identify patients most suitable for comprehensive geriatric assessment.

Screening for frailty may act to decrease age related discrimination by identifying robust elderly patients. Screening can also identify the most frail and trigger discussions regarding limitations of treatment.

Other studies have looked at the CFS as a predictor of mortality in the acute hospital setting [11–13, 15, 17]. This study also examines functional outcomes. Failure to return to pre-morbid functional status predicts mortality [2] and institutionalization [21]. Patients who are at high risk for functional decline are also at high risk for mortality [2].

There was no association found between length of stay and frailty score, which is not consistent with other studies [15, 17]. This may be due to the high prevalence of frailty, meaning that discrimination was lost, as other studies that have examined this association have had lower proportions of frail patients [3]. In the studies by Wallis et al. [17] and Evans et al. [3] it is not clear whether length of stay included subacute care, which was not included in our study.

There is a positive relationship between the degree of frailty and the risk of mortality and functional decline when frailty is measured by FI-CGA [3]. This has also been demonstrated with the CFS in other studies [15].

Consistent with previous findings, female gender conferred protection against mortality [22].

Wallis et al. conducted a retrospective study to determine the association of the CFS with patient characteristics and outcomes. The CFS was completed for all patients aged 75 or older as part of routine care by junior medical or nursing staff, who were provided with training at induction. Despite the lack of training provided for the junior medical staff in our study, the OR for inpatient mortality was 1.6, 95 % CI 1.48 1.74, which was comparable to the three month mortality rate in our study of 1.82, 95 % CI 1.14, 2.91. Similar to our findings, Wallis et al. [17] demonstrated that the least frail patients had slightly higher mortality than the moderately frail patients. This may be due to patients who are more robust only needing to be hospitalized for more a more severe interceding illness. In a similar population to ours, Basic and Shanley [15] also found a higher risk of mortality with increasing frailty identified on the CFS.

The study has certain strengths. A high proportion of eligible patients were included. Since individual consent/assent was not required and the CFS could be completed at any time during the hospital stay there were no barriers to recruitment of patients with communication, language or cognitive difficulties or those admitted outside routine working hours. This increases the generalisability of our findings to patients who have barriers to communication.

We also acknowledge methodological weaknesses. This is a single centre study, and so the results may not be applicable to other sites.

The measure of functional decline was indirect, as it was the need for subacute care, the need for increased services on discharge or the opinion of an allied health team member that the patient was below pre-morbid function. As we relied on routine clinical data, there was no direct measurement tool available. Although this is not a validated measure, the proportion of patients who experienced functional decline was similar to other studies in similar settings [2, 23]. Other studies have used a count of activities of daily living and instrumental activities of daily living as a marker of functional decline [24], and this is similar to the assessment performed by physiotherapists and occupational therapists. A new need for residential care has also been used as a marker of functional decline [25]. In this hospital setting it is rare for a patient to be newly discharged to residential care without being admitted to subacute care, so this was deemed a more appropriate measure.

Patients who were from residential care were excluded from the analysis of functional decline, as some of the criteria used to define functional decline were not applicable to this group. The measure used may have lacked sufficient sensitivity to detect functional decline in those who already had low baseline function, for example people receiving full time care from family members. These limitations could be overcome by conducting further research with an objective measure of function at the time of hospitalization and the time of discharge.

We were unable to include some potential confounders in the multivariate analysis. Only information that was routinely collected for patients as part of standard medical, allied health and nursing care was available, so we were unable to obtain a measure of nutrition, cognition or delirium.

As this study was conducted in a real world setting, we were unable to obtain inter-rater reliability. The CFS has previously been demonstrated to have high inter-rater reliability [11, 26].

A general limitation of frailty measurement in the acute setting, it that it is possible that the level of frailty is over-stated due to the effect of the antecedent illness. Many patients experience functional decline prior to hospital admission [27], which will lead to a higher frailty score. If the antecedent insult (such as infection, new drug) causes a functional decline resulting in a higher frailty score this still may represent a bad prognostic factor. The only way to examine this would be to look at prospective cohorts recruited in the community.

## Conclusions

It is increasingly recognised that frailty rather than chronological age predicts adverse outcomes in hospitalized older adults. Identification of frailty in the acute setting within time and resource limitations is a major challenge. The CFS is quick and easy to use, and acceptable to busy junior clinicians. This trial demonstrates the feasibility of using the CFS in the acute setting. Incorporating this into routine care has the potential to improve the recognition and measurement of frailty. Future research should investigate how the CFS correlates with more precise measures of frailty, particularly the frailty index derived from comprehensive geriatric assessment and if multifaceted interventions including management of cognition, nutrition and social factors can improve outcomes for patients with different levels of frailty.

## Abbreviations

CFS, clinical frailty scale; CGA, comprehensive geriatric assessment; FI-CGA, frailty index based on a comprehensive geriatric assessment.
